# Conventional ultrasound and contrast-enhanced ultrasound radiomics in breast cancer and molecular subtype diagnosis

**DOI:** 10.3389/fonc.2023.1158736

**Published:** 2023-05-23

**Authors:** Xuantong Gong, Qingfeng Li, Lishuang Gu, Chen Chen, Xuefeng Liu, Xuan Zhang, Bo Wang, Chao Sun, Di Yang, Lin Li, Yong Wang

**Affiliations:** ^1^ Department of Ultrasound, National Cancer Center/National Clinical Research Center for Cancer/Cancer Hospital, Chinese Academy of Medical Sciences and Peking Union Medical College, Beijing, China; ^2^ School of Computer Science and Engineering, Beihang University, Beijing, China; ^3^ Department of Ultrasound, Beijing Hospital of Traditional Chinese Medicine, Capital Medical University, Beijing, China; ^4^ Hangzhou Innovation Institute, Beihang University, Hangzhou, China; ^5^ State Key Laboratory of Virtual Reality Technology and Systems, School of Computer Science and Engineering, Beijing Advanced Innovation Center for Big Data and Brain Computing (BDBC), Beihang University, Beijing, China; ^6^ Department of Ultrasound, Dongzhimen Hospital, Beijing University of Chinese Medicine, Beijing, China; ^7^ Department of Diagnostic Radiology, National Cancer Center/National Clinical Research Center for Cancer/Cancer Hospital, Chinese Academy of Medical Sciences and Peking Union Medical College, Beijing, China

**Keywords:** radiomics, ultrasound, contrast-enhanced ultrasound, breast cancer, molecular subtype

## Abstract

**Objectives:**

This study aimed to explore the value of conventional ultrasound (CUS) and contrast-enhanced ultrasound (CEUS) radiomics to diagnose breast cancer and predict its molecular subtype.

**Method:**

A total of 170 lesions (121 malignant, 49 benign) were selected from March 2019 to January 2022. Malignant lesions were further divided into six categories of molecular subtype: (non-)Luminal A, (non-)Luminal B, (non-)human epidermal growth factor receptor 2 (HER2) overexpression, (non-)triple-negative breast cancer (TNBC), hormone receptor (HR) positivity/negativity, and HER2 positivity/negativity. Participants were examined using CUS and CEUS before surgery. Regions of interest images were manually segmented. The pyradiomics toolkit and the maximum relevance minimum redundancy algorithm were utilized to extract and select features, multivariate logistic regression models of CUS, CEUS, and CUS combined with CEUS radiomics were then constructed and evaluated by fivefold cross-validation.

**Results:**

The accuracy of the CUS combined with CEUS model was superior to CUS model (85.4% vs. 81.3%, p<0.01). The accuracy of the CUS radiomics model in predicting the six categories of breast cancer is 68.2% (82/120), 69.3% (83/120), 83.7% (100/120), 86.7% (104/120), 73.5% (88/120), and 70.8% (85/120), respectively. In predicting breast cancer of Luminal A, HER2 overexpression, HR-positivity, and HER2 positivity, CEUS video improved the predictive performance of CUS radiomics model [accuracy=70.2% (84/120), 84.0% (101/120), 74.5% (89/120), and 72.5% (87/120), p<0.01].

**Conclusion:**

CUS radiomics has the potential to diagnose breast cancer and predict its molecular subtype. Moreover, CEUS video has auxiliary predictive value for CUS radiomics.

## 1 Introduction

Breast cancer is the most common malignant tumor worldwide and is the leading cause of cancer-related death in women (1). In 2011, according to the immunohistochemical detection of estrogen receptor (ER) and progesterone receptor (PR), as well as human epidermal growth factor receptor 2 (HER2) and Ki-67 labeling index, St. Gallen’s expert panel proposed the classification of breast cancer into four subtypes: Luminal A, Luminal B, HER2 overexpression, and basal-like subtypes (2). The treatment plan and the prognosis of patients with different subtypes are different (3, 4). Therefore, the accurate diagnosis of breast cancer molecular subtypes is of great significance for guiding doctors to develop individualized treatment plans.

Medical imaging examination is a repeatable and non-invasive method to obtain comprehensive tumor information (5). Radiomics is an effective imaging analysis method that utilizes computer algorithms to extract quantitative features from medical images (computed tomography [CT], magnetic resonance imaging [MRI], ultrasound [US], etc.) in a high-throughput way and transform traditional images into mineable high-dimensional data (6). Radiomics can deeply excavate image information that cannot be distinguished by visual analysis, and combine this information with the clinical pathological information of patients to develop a model to improve the diagnostic accuracy and evaluate the prognosis of diseases (7–9). Radiomics assumes that the extracted data are the product of tumor development at the genetic and molecular levels and may be related to the biological behavior of the tumor and the prognosis of patients (10, 11), which provides a research basis for radiomics in the study of molecular subtype of breast cancer. Radiomics provides a noninvasive and comprehensive evaluation of tumors compared to puncture.

Conventional ultrasound (CUS) is one of the main examination methods for the diagnosis of breast cancer. Compared to CUS, contrast-enhanced ultrasound (CEUS) can not only reflect the morphological characteristics of the tumor but also reveal the perfusion of tumor microcirculation continuously and dynamically which can provide more extra information (12, 13). Studies have shown that CEUS performance in breast cancer has a certain correlation with its prognostic factors, and the visualized structure of tumor microcirculation provides valuable information for predicting the molecular subtype of breast cancer (14).

Currently, MRI, mammography (MG), and positron emission tomography-CT (PET-CT) radiomics have been proven to be significant in the diagnosis and molecular subtype prediction of breast cancer (15–18). However, based on CUS image and CEUS video radiomics, there are rare studies on the diagnosis of breast cancer and a lack of research on the prediction of molecular subtype of breast cancer. We aimed to explore the value of CUS combined with CEUS radiomics in diagnosing breast cancer and predicting the molecular subtype of breast cancer.

## 2 Materials and methods

### 2.1 Study participants

**Figure 1:**
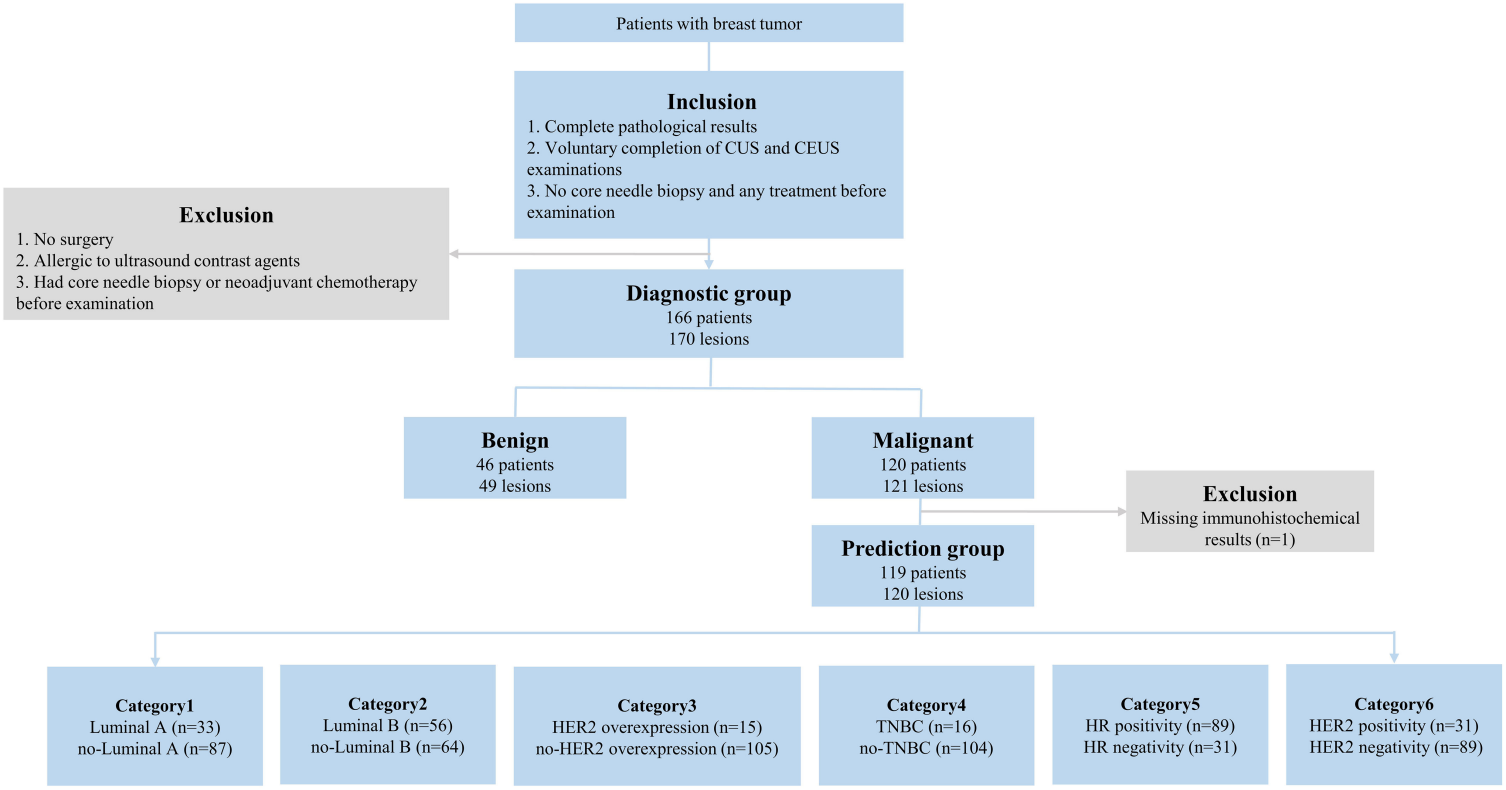
The inclusion/exclusion flow chart. n = number of lesions. Luminal A: ER and/or PR+, HER2-, Ki-67<14%; Luminal B (HER2-): ER and/or PR+, HER2-, Ki-67≥14%; Luminal B (HER2+): ER and/or PR+, HER2+, any Ki-67; HER2 overexpression: ER and/or PR-, HER2+. TNBC: ER-, PR-, HER2-. CUS, conventional ultrasound; CEUS, contrast-enhanced ultrasound; ER, estrogen receptor; PR, progesterone receptor; HER2, human epidermal growth factor receptor 2; TNBC, triple-negative breast cancer; HR, hormone receptor.

This prospective study was approved by the Institutional Ethics Board (NCC2019C-178) and all patients signed informed consent forms. From March 2019 to January 2022, 166 patients (170 lesions) with complete pathological results, no core needle biopsy, and any treatment before the examination, completion of CUS and CEUS examinations were selected into the diagnostic group. Among them, 119 participants (120 lesions) with postoperative pathological results were finally selected for the prediction group (**Figure 1**). The participants were examined by CUS and CEUS before the operation.

### 2.2 CUS of breast

Two senior sonographers used Philips EPIQ5 ultrasonic diagnostic equipment (Philips, Bothell, WA), selected high-frequency linear array probes, focused on the center of the tumor, and collected all images containing the CUS characteristics of the tumor.

### 2.3 CEUS of breast

Using the same ultrasonic diagnostic equipment, adjusted the image to show the larger section of the tumor clearly, switched to the real-time imaging condition of the double-frame CEUS mode, and made the single point focus on the deepest part of the image. Then, 4.8 mL of the contrast agent (SonoVue) was drawn and injected into the patient’s pre-established peripheral venous channel, followed by 5 mL of saline. Meanwhile, started the timer and image storage function, and observed the perfusion of tumor microcirculation for 3 minutes.

### 2.4 Pathological examination

All breast tumors were surgically removed. According to immunohistochemistry analysis results and expert consensus on the molecular subtype of breast cancer at the St. Gallen’s meeting in 2013 (19), lesions of breast cancer were first classified into four categories of Luminal A and non-Luminal A, Luminal B and non-Luminal B, HER2 overexpression and non-HER2 overexpression, and triple-negative breast cancer (TNBC) and non-TNBC. Extra two categories of HR positivity/negativity and HER2 positivity/negativity were added to this study due to their high guiding value in clinical treatment.

### 2.5 Image/video segmentation

**Figure 2:**
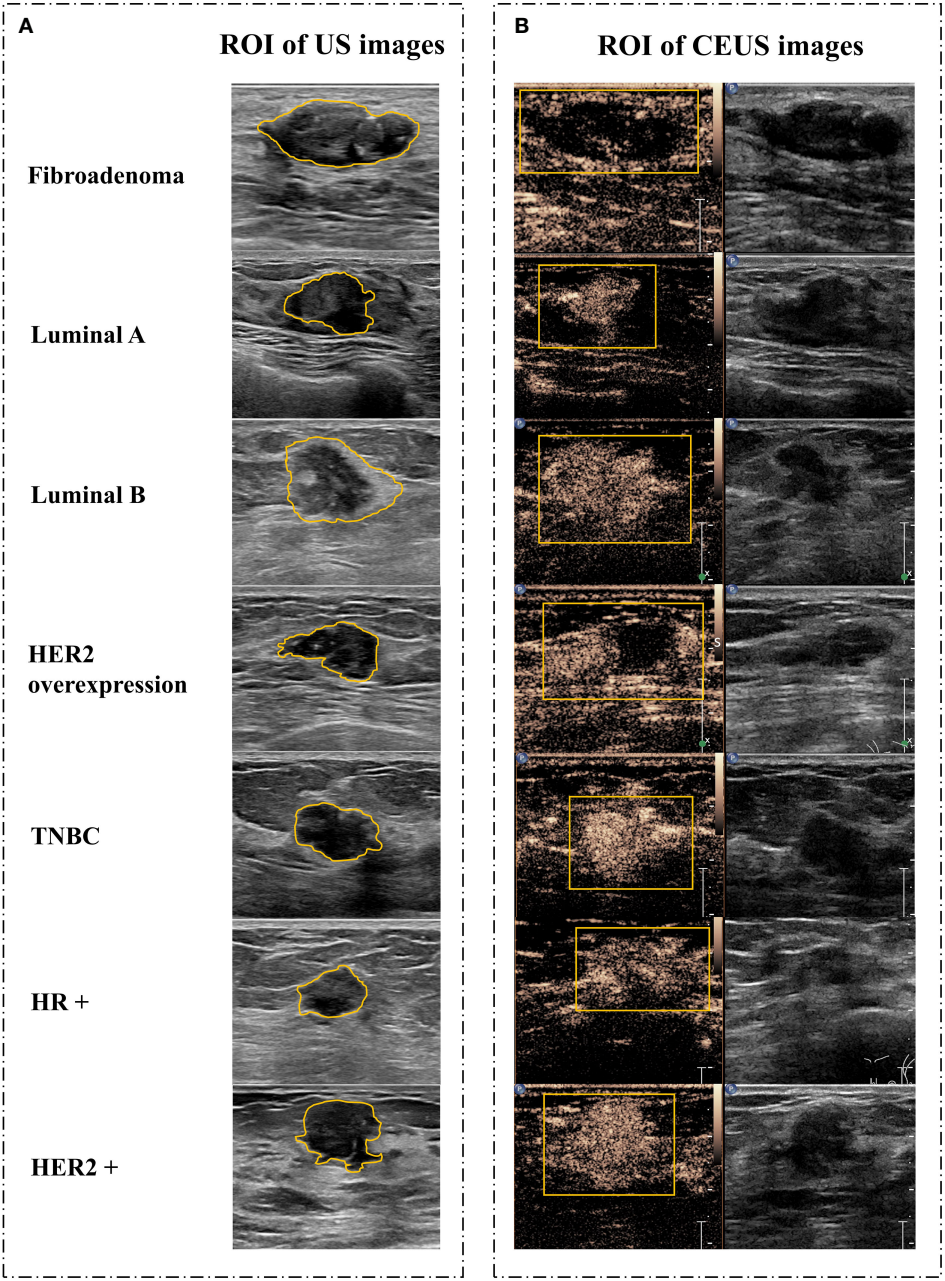
Image segmentation. **(A)** Schematic diagram of CUS images. **(B)** Schematic diagram of corresponding CEUS images with the peak intensity. CUS, conventional ultrasound; CEUS, contrast-enhanced ultrasound; ROI, region of interest; HER2, human epidermal growth factor receptor 2; TNBC, triple-negative breast cancer; HR, hormone receptor.

There were 967 images of CUS, and 170 videos of CEUS in total. The region of interest (ROI) sketch was drawn manually by two senior physicians and areas with disagreement were jointly assessed. The ROI of CUS images was outlined around the tumor, including the hyperechoic halo. For the ROI of CEUS videos, it was first outlined with a rectangular box in the frame that the tumor displayed clearly (**Figure 2**), and then, a computer vision algorithm was used to track and draw all the ROI sketches across all the frames in the video (20).

### 2.6 Radiomics feature extraction and analysis

Shape features, first-order statistical features, and texture features were extracted using the pyradiomics toolkit (https://github.com/Radiomics/pyradiomics) from original images and 14 transformed images, respectively. The transformed images were generated by performing 2D discrete wavelet decomposition and reconstruction or filtering by the Laplacian of Gaussian method with different sigma parameters. Shape features of CEUS images were removed during feature selection because the usage of the rectangular box could not delineate the ROI with correct shape information. For multiple images of each lesion, the maximum value of the feature was taken as the final characteristic value.

### 2.7 Construction of the diagnostic and prediction model

The mRMR algorithm was used to select the most effective feature subsets, out of the diagnostic group and six categories of breast cancer (21). Then, based on the selected feature subset, multivariate logistic regression models of CUS, CEUS, and CUS combined with CEUS were constructed in the diagnostic group and six categories of breast cancer.

The performance of models was evaluated by fivefold cross-validation. The data were divided into five independent data sets equally, four of which were used to train the model, and the other one was used as an independent validation set to obtain predictive results. Finally, in a round of fivefold training, all the samples were used once to validate the model performance. The aforementioned experiment was conducted 100 times with different random seeds to further enhance the reliability of the model’s prediction results. The average value of the corresponding indicators was used as the final prediction result. The receiver operator characteristic (ROC) curve, the area under the curve (AUC), 95% confidence interval (CI), Youden index (YI), and the corresponding cut-off values were used for training evaluation. Sensitivity (SE), specificity (SP), accuracy (ACC), positive predictive value (PPV), and negative predictive value (NPV) of the model were presented to describe the model performance.

### 2.8 Statistical analysis

SPSS 23.0 software (SPSS Inc., Chicago, IL, USA) and Python3.6 software (http://www.python.org) were used for data analyses. Enumeration data were expressed in percentage (%), and the difference between groups was compared with a chi-square test. Measurement data are expressed as mean ± standard deviation. The difference between groups was first analyzed using the K-S test to analyze the normality of the data distribution. Then, for normally distributed data, an independent-samples t-test was adopted, while the Mann-Whitney U test was used for non-normally distributed data. P < 0.05 was used as the significant standard for all the above statistical tests.

## 3 Results

### 3.1 Clinicopathological data

In the diagnostic group, 166 women were enrolled, and most of the participants were diagnosed with a breast mass (90.96%, 151/166), followed by nipple discharge (6.03%, 10/166) and breast calcification (3.01%, 5/166). A total of 170 lesions were included (49 benign lesions and 121 malignant lesions). Benign lesions had 19 cases of fibroadenoma, 15 cases of intraductal papilloma, 12 cases of adenopathy, 2 cases of ductal epithelial hyperplasia, and 1 case of fat necrosis with abscess formation. Malignant lesions had 85 cases of invasive carcinoma with no specific type, 17 cases of ductal carcinoma *in situ* with partial invasive carcinoma, 11 cases of ductal carcinoma *in situ*, 3 cases of papillary carcinoma, 2 cases of invasive lobular carcinoma, 2 cases of mucinous carcinoma and 1 case of phyllodes tumor. The average age of patients with benign lesions was 44.26 ± 8.94 years (range 23–64 years), and the average age of patients with malignant lesions was 51.48 ± 10.41 years (range 32–85 years).

In the prediction group, 119 women were enrolled, with an average age of 51.56 ± 10.33 years (range 32–85 years). Most of the participants were diagnosed with a breast mass (94.1%, 112/119), followed by nipple discharge (3.4%, 4/119) and breast calcification (2.5%, 3/119). Because of the lack of immunohistochemistry results for one case of a phyllodes tumor, a total of 120 lesions of cancer were finally selected, including 33 cases of Luminal A, 56 cases of Luminal B (16 cases of HER2-positivity and 40 cases of HER2-negativity), 15 cases of HER2 overexpression, and 16 cases of TNBC. In addition, there were 89 cases of HR-positivity breast cancer and 31 cases of HER2-positivity breast cancer.

A comparison of clinicopathological data of benign and malignant breast tumors was presented in **Table 1**. The mean age of patients in the malignant group was higher than that in the benign group, the maximum and minimum diameters of breast cancer were larger than that of the benign tumor (p<0.05). A comparison of clinicopathological data in different categories of breast cancer was presented in **Table 2**. The maximum and minimum diameters in the Luminal A group were smaller than those in the non-Luminal A group (p=0.002 and p=0.004). Additionally, the maximum diameter in the HER2-overexpression group was larger than that in the non-HER2-overexpression group (p=0.023), and the maximum diameter in the HR-negativity group was larger than that in the HR-positivity group (p=0.009).

**Table 1:** Comparison of clinicopathological data of benign and malignant breast tumor.

	Benign	Malignancy	z/χ2 value	p value
**Age (years)**	44.26 ± 8.94	51.48 ± 10.41	-4.18	<0.05*
**Clinical manifestation**			5.13	0.08
Breast mass	39 (82.98%)	112 (94.12%)		
Nipple discharge	6 (12.77%)	4 (3.36%)		
Breast calcification	2 (4.25%)	3 (2.52%)		
Pathological result				
Fibroadenoma	19 (38.78%)	\		
Intraductal papilloma	15 (30.61%)	\		
Adenopathy	12 (24.49%)	\		
Ductal epithelial hyperplasia	2 (4.08%)	\		
Fat necrosis with abscess formation	1 (2.04%)	\		
Invasive carcinoma with no specific type	\	85 (70.25%)		
Ductal carcinoma *in situ* with partial invasive carcinoma	\	17 (14.05%)		
Ductal carcinoma *in situ*	\	11 (70.25%)		
Papillary carcinoma	\	3 (9.09%)		
Invasive lobular carcinoma	\	2 (1.65%)		
Mucinous carcinoma	\	2 (1.65%)		
Phyllodes tumor	\	1 (0.83%)		
**Maximum diameter (cm)**	1.31 ± 0.78	2.17 ± 1.15		<0.05*
**Minimum diameter (cm)**	0.86 ± 0.48	1.43 ± 0.94		<0.05*

* The difference was statistically significant(p<0.05)

**Table 2:** Comparison of clinicopathological data in different categories of breast cancer.

	Group		z/t value	p value
Category 1	Luminal A	non-Luminal A		
Number of lesions	33	87		
Age	51.697 ± 11.246	51.506 ± 10.023	0.187^a^	0.852
Maximum diameter (cm)	1.667 ± 0.859	2.320 ± 1.151	-3.109	0.002*
Minimum diameter (cm)	1.133 ± 0.773	1.505 ± 0.928	-2.09	0.004*
Category 2	Luminal B	non-Luminal B		
Number of lesions	56	64		
Age	51.857 ± 10.721	51.297 ± 10.044	0.295^a^	0.768
Maximum diameter (cm)	2.107 ± 0.896	2.169 ± 1.281	-0.506	0.613
Minimum diameter (cm)	1.423 ± 0.806	1.384 ± 0.982	-1.015	0.310
Category 3	HER2-overexpression	non-HER2-overexpression		
Number of lesions	15	105		
Age	50.200 ± 7.282	51.752 ± 10.703	0.543^a^	0.588
Maximum diameter (cm)	2.660 ± 1.151	2.066 ± 1.094	-2.266	0.023*
Minimum diameter (cm)	1.547 ± 0.808	1.382 ± 0.915	-1.312	0.190
Category 4	TNBC	non-TNBC		
Number of lesions	16	104		
Age	51.500 ± 10.139	51.567 ± 10.402	0.024^a^	0.981
Maximum diameter (cm)	2.743 ± 1.713	2.047 ± 0.970	-1.137	0.256
Minimum diameter (cm)	1.750 ± 1.362	1.349 ± 0.803	-1.055	0.291
Category 5	HR+	HR-		
Number of lesions	89	31		
Age	51.798 ± 10.856	50.871 ± 8.751	-0.429^a^	0.669
Maximum diameter (cm)	1.944 ± 0.903	2.703 ± 1.445	-2.595	0.009*
Minimum diameter (cm)	1.316 ± 0.802	1.652 ± 1.115	-1.810	0.070
Category 6	HER2+	HER2-		
Number of lesions	31	89		
Age	50.710 ± 7.493	51.854 ± 11.167	-0.638^a^	0.525
Maximum diameter (cm)	2.245 ± 0.966	2.103 ± 1.164	-1.174	0.242
Minimum diameter (cm)	1.436 ± 0.695	1.391 ± 0.965	-1.341	0.128

a : t value; HER2, human epidermal growth factor receptor 2; TNBC, triple-negative breast cancer; HR, hormone receptor; * The difference was statistically significant(p<0.05).

### 3.2 Evaluation of diagnostic models

**Figure 3:**
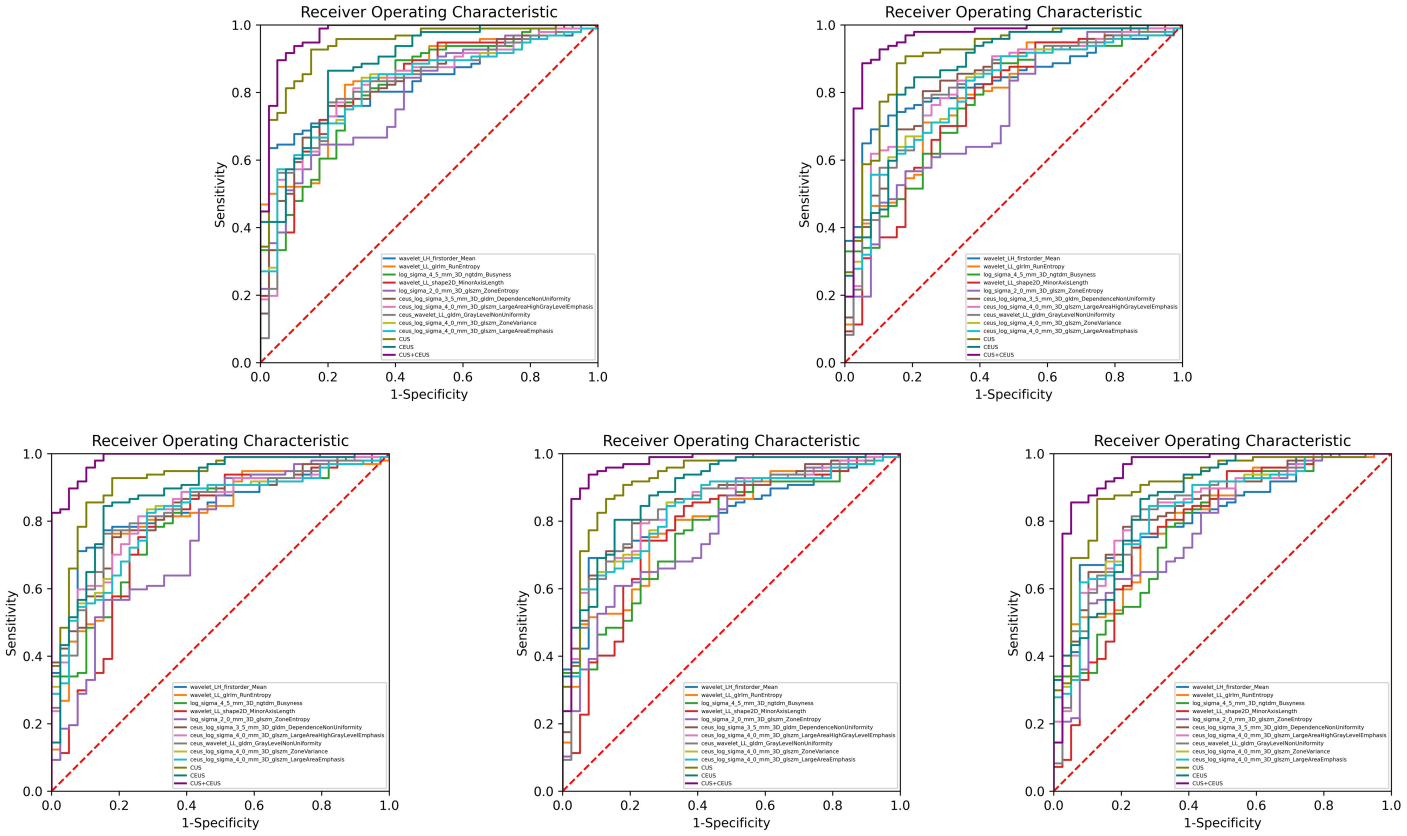
Training ROC curves of diagnostic group. Extracted from one round of fivefold cross training. As shown, CUS combined with CEUS radiomics model performed the best, and all the 3 constructed models were better than the single feature. CUS, conventional ultrasound; CEUS, contrast-enhanced ultrasound.

The performance of diagnostic models was shown in **Table 3**, and one round training ROC curve was shown in **Figure 3**.

**Table 3:** Results of constructed radiomics models in diagnosing breast cancer.

Diagnostic model	AUC (95%CI)	YI	Cut-off	SE (95%CI)	SP (95%CI)	ACC (95%CI)	PPV (95%CI)	NPV (95%CI)
CUS	0.919	0.732	0.625	84.4%	73.7%	81.3%	88.8%	65.7%
	(0.896,0.937)			(76.9%,89.8%)	(59.9%,83.9%)	(74.8%,86.5%)	(81.7%,93.3%)	(52.5%,76.9%)
CEUS	0.872	0.631	0.674	81.3%	72.1%	78.6%	87.8%	60.9%
	(0.844,0.895)			(73.4%,87.2%)	(58.3%,82.7%)	(71.8%,84.1%)	(80.4%,92.6%)	(48.0%,72.4%)
CUS+CEUS	0.953	0.814	0.605	87.5%	80.2%	85.4%	91.6%	72.2%
	(0.935,0.967)			(80.4%,92.3%)	(67.1%,89.0%)	(79.3%,89.9%)	(85.1%,95.4%)	(59.1%,82.3%)

CUS, conventional ultrasound; CEUS, contrast-enhanced ultrasound; AUC, area under the curve; YI, Youden index; CI, confidence interval; SE, sensitivity; SP, specificity; ACC, accuracy; PPV, positive predictive value; NPV, negative predictive value.

CUS radiomics model achieved an AUC of 0.919 (95% CI: 0.896, 0.937) in the training set. In the validation set, the ACC of the model was 81.3% (138/170), SE was 84.4% (102/121), SP was 73.7% (36/49), PPV was 88.8% (102/115), and NPV was 65.7% (36/55).

CEUS radiomics model achieved an AUC of 0.872 (95% CI: 0.844, 0.895) in the training set. In the validation set, the ACC of the model was 78.6% (134/170), SE was 81.3% (98/121), SP was 72.1% (35/49), PPV was 87.8% (98/112), and NPV was 60.9% (35/58).

CUS combined with CEUS radiomics model achieved an AUC of 0.953 (95% CI: 0.935, 0.967) in the training set. In the validation set, the ACC of the model was 85.4% (145/170), SE was 87.5% (106/121), SP was 80.2% (39/49), PPV was 91.6% (106/116), and NPV was 72.2% (39/54). The diagnostic ACC of the combined model was significantly higher than that of the CUS model (p<0.001).

### 3.3 Evaluation of prediction models

**Figure 4:**
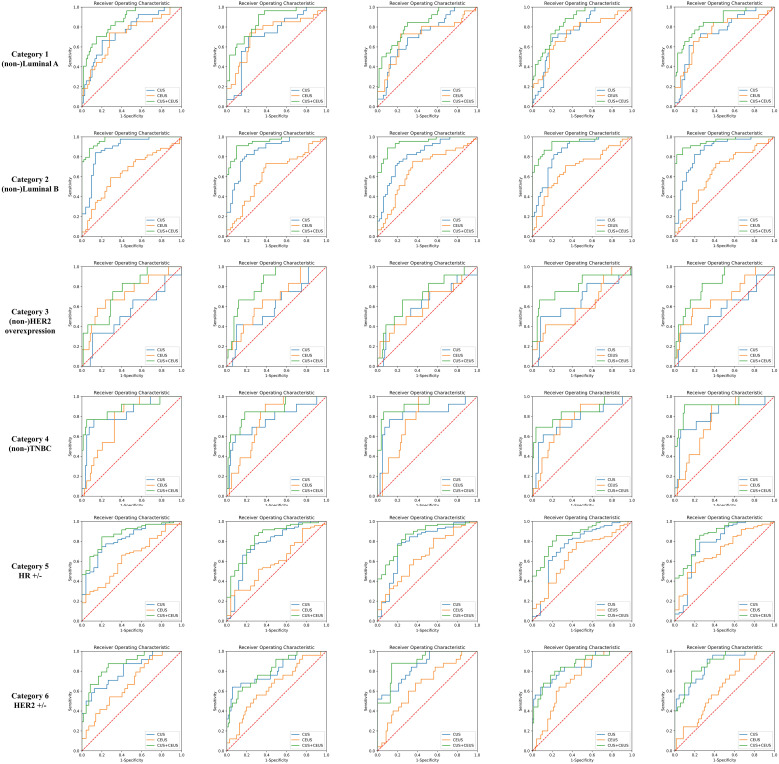
Training ROC curves of prediction group. Extracted from one round of fivefold cross training in each category. As shown, CUS combined with CEUS radiomics model achieved better performances. CUS, conventional ultrasound; CEUS, contrast-enhanced ultrasound; HER2, human epidermal growth factor receptor 2; TNBC, triple-negative breast cancer; HR, hormone receptor.

The performance of prediction models in the six categories was shown in **Table 4**, and one round training ROC curve was shown in **Figure 4**.

**Table 4:** Results of constructed radiomics models in predicting different categories of breast cancer.

Predictive model	AUC (95%CI)	YI	Cut-off	SE (95%CI)	SP (95%CI)	ACC (95%CI)	PPV (95%CI)	NPV (95%CI)
Category 1								
CUS	0.726	0.436	0.300	53.2%	73.8%	68.2%	43.6%	80.6%
	(0.685,0.764)			(36.8%,69.0%)	(63.7%,81.9%)	(59.4%,75.8%)	(29.5%,58.8%)	(70.6%,87.8%)
CEUS	0.738	0.457	0.299	55.7%	74.9%	69.6%	45.7%	81.7%
	(0.697,0.776)			(39.1%,71.2%)	(64.9%,82.8%)	(60.9%,77.1%)	(31.4%,60.8%)	(71.8%,88.6%)
CUS+CEUS	0.730	0.461	0.323	58.0%	74.9%	70.2%*	46.7%	82.5%
	(0.688,0.767)			(41.2%,73.1%)	(64.9%,82.8%)	(61.5%,77.7%)	(32.4%,61.6%)	(72.6%,89.3%)
Category 2								
CUS	0.840	0.593	0.452	71.3%	67.6%	69.3%	65.8%	72.9%
	(0.805,0.870)			(58.4%,81.5%)	(55.4%,77.8%)	(60.6%,76.9%)	(53.3%,76.5%)	(60.5%,82.6%)
CEUS	0.671	0.357	0.491	61.3%	63.5%	62.5%	59.5%	65.2%
	(0.628,0.712)			(48.2%,72.9%)	(51.3%,74.3%)	(53.6%,70.6%)	(46.6%,71.2%)	(52.8%,75.8%)
CUS+CEUS	0.888	0.672	0.481	69.2%	70.2%	69.7%	67.0%	72.2%
	(0.857,0.913)			(56.2%,79.7%)	(58.1%,80.0%)	(61.0%,77.2%)	(54.1%,77.7%)	(60.1%,81.8%)
Category 3								
CUS	0.605	0.321	0.165	32.1%	91.0%	83.7%	33.8%	90.4%
	(0.561,0.648)			(14.4%,57.2%)	(84.0%,95.1%)	(76.0%,89.2%)	(15.2%,59.3%)	(83.3%,94.7%)
CEUS	0.612	0.334	0.160	20.2%	86.8%	78.4%	17.9%	88.4%
	(0.568,0.655)			(7.2%,45.4%)	(79.0%,92.0%)	(70.3%,84.9%)	(6.3%,41.4%)	(80.8%,93.2%)
CUS+CEUS	0.652	0.393	0.174	34.2%	91.2%	84.0%*	35.6%	90.7%
	(0.608,0.693)			(15.8%,59.1%)	(84.2%,95.2%)	(76.5%,89.5%)	(16.5%,60.8%)	(83.6%,94.9%)
Category 4								
CUS	0.803	0.596	0.234	60.4%	90.8%	86.7%	50.2%	93.7%
	(0.765,0.836)			(36.8%,80.0%)	(83.7%,95.0%)	(79.5%,91.7%)	(29.8%,70.6%)	(87.2%,97.0%)
CEUS	0.749	0.521	0.176	54.0%	80.8%	77.2%	30.2%	91.9%
	(0.709,0.7867)			(31.3%,75.2%)	(72.2%,87.2%)	(69.0%,83.8%)	(16.6%,48.5%)	(84.5%,96.0%)
CUS+CEUS	0.829	0.632	0.251	58.4%	91.3%	86.9%	50.8%	93.4%
	(0.793,0.860)			(35.0%,78.5%)	(84.3%,95.4%)	(79.7%,91.8%)	(29.9%,71.5%)	(86.9%,96.8%)
Category 5								
CUS	0.817	0.565	0.709	78.1%	60.3%	73.5%	84.9%	49.0%
	(0.780,0.849)			(68.5%,85.5%)	(42.8%,75.4%)	(65.0%,80.6%)	(75.7%,91.1%)	(33.9%,64.2%)
CEUS	0.743	0.446	0.693	69.8%	46.3%	63.7%	78.9%	34.8%
	(0.702,0.780)			(59.6%,78.4%)	(30.2%,63.3%)	(54.8%,71.8%)	(68.6%,86.4%)	(22.2%,50.1%)
CUS+CEUS	0.855	0.626	0.703	78.8%	62.1%	74.5%*	85.6%	50.5%
	(0.821,0.884)			(69.2%,86.0%)	(44.6%,76.9%)	(66.0%,81.4%)	(76.5%,91.6%)	(35.3%,65.6%)
Category 6								
CUS	0.837	0.552	0.351	45.6%	79.6%	70.8%	43.8%	80.8%
	(0.801,0.867)			(29.6%,62.6%)	(70.1%,86.7%)	(62.2%,78.2%)	(28.3%,60.7%)	(71.3%,87.7%)
CEUS	0.665	0.358	0.287	38.5%	76.3%	66.6%	36.2%	78.1%
	(0.621,0.706)			(23.5%,55.9%)	(66.5%,84.0%)	(57.7%,74.4%)	(22.0%,53.2%)	(68.3%,85.5%)
CUS+CEUS	0.960	0.823	0.383	46.5%	81.6%	72.5%*	46.8%	81.4%
	(0.938,0.974)			(30.3%,63.4%)	(72.3%,88.3%)	(63.9%,79.7%)	(30.5%,63.8%)	(72.1%,88.1%)

**Category1:** (non-)Luminal A; **Category2:** (non-)Luminal B; **Category3:** (non-)human epidermal growth factor receptor 2 (HER2) overexpression; **Category4:** (non-)triple-negative breast cancer (TNBC); **Category5:** hormone receptor (HR) positivity/negativity; **Category 6:** HER2 positivity/negativity categories.

CUS, conventional ultrasound; CEUS, contrast-enhanced ultrasound; AUC, area under the curve; YI, Youden index; CI, confidence interval; SE, sensitivity; SP, specificity; ACC, accuracy; PPV, positive predictive value; NPV, negative predictive value.

* The performance improvement of CEUS was statistically significant (p<0.05).

The ACCs of CUS radiomics model for predicting the 6 categories of breast cancer were 68.2% (82/120), 69.3% (83/120), 83.7% (100/120), 86.7% (104/120), 73.5% (88/120), and 70.8% (85/120), respectively. The ACCs of CUS combined with CEUS radiomics model were 70.2% (84/120), 69.7% (83/120), 84.0% (101/120), 86.9% (104/120), 74.5% (89/120), and 72.5% (87/120), respectively. CEUS radiomics significantly improved the performance of CUS radiomics in predicting Luminal A, HER2-overexpression, HR-positivity, and HER2-positivity breast cancers subtype (p=0.000013, 0.003, 0.004, and 0.000145).

## 4 Discussion

To our knowledge, this is the first time that CEUS videos were combined with CUS images to construct the radiomics model to diagnose breast cancer and also predict its molecular subtype. We found that CEUS videos that contained tumor microcirculation perfusion can improve the performance of CUS radiomics models, the combined model achieved excellent diagnostic results (AUC=0.953, ACC=85.4%). Further, we revealed the feasibility of CUS combined with CEUS radiomics model in predicting molecular subtype of breast cancer before surgery, which has important guiding value for clinical treatment.

Studies have shown that CUS radiomics has diagnostic value in the differentiation of benign and malignant breast tumors (8, 22, 23). Li et al. selected the features of 181 breast lesions (67 malignant and 114 benign) and found that the ACC of CUS combined with CEUS radiomics model was 75.0% (AUC=0.873), higher than that in the CUS radiomics model (ACC=67.6%, AUC=0.767) (24). In our study, the constructed CUS combined with CEUS model was also better than the CUS model. The reason could be that the extra dynamic blood perfusion information of the tumor in CEUS videos contributed to diagnosing breast cancer. Besides, because of the difference in ROI delineation methods, the CEUS features had comparative information between the tumor and its surrounding tissues which had value for the diagnosis results (25, 26).

This study demonstrated that CUS radiomics has the power in predicting the molecular subtype of breast cancer, which has also been proven in previous studies (27, 28). Guo et al. developed an automated model of radiomics to evaluate the correlation between CUS radiomics features and the biological characteristics of invasive ductal carcinoma. They found that AUC, SE, and SP of the model distinguished between breast cancer of HR-positivity with HER2-negativity and TNBC were 0.760 (95%CI: 0.755, 0.764), 97.9%, and 60.1%, respectively (27). Yu et al. found that the CUS radiomics nomogram had a moderate predictive ability for axillary lymph node metastasis of invasive breast cancer before surgery, in which the AUC (0.84 (95%CI: 0.80, 0.89)) was better than the clinical model (AUC=0.76 (95%CI: 0.71, 0.82), p<0.001) and direct CUS evaluation (AUC=0.70 (95%CI: 0.64, 0.76), p<0.001) (28). Currently, radiomics based on MRI, MG, and PET-CT images has made progress in predicting the molecular subtype and biological characteristics of breast cancer. They are being increasingly used in contrast-enhanced images with tumor blood perfusion (16, 18, 29). Based on CUS image, CEUS video can additionally reflect the perfusion of tumor microcirculation and provide more biological information about the tumor. We found the significant auxiliary value of CEUS radiomics to CUS radiomics in the prediction of Luminal A, HER2 overexpression, HR-positivity, and HER2-positivity breast cancer. This could be due to the different biological performances of different subtypes of breast cancer. Luminal A breast cancer is mostly highly differentiated and has lower expression of tumor cell proliferation markers and slower growth. Most cases of HER2-positivity breast cancer and TNBC are poorly differentiated with a high degree of malignancy, strong aggressiveness, rapid growth, and prone to necrosis of the internal tissues of the tumor (15, 30, 31). It is, therefore, easier to find the real necrotic area using CEUS video; hence, the texture features of CEUS radiomics can reflect the internal differences of tumors that are indistinguishable by eyes and assist in the diagnosis of CUS. Some studies have also shown that ER-negative breast cancer has a higher expression level of vascular endothelial growth factor, thus showing higher microvascular density and more apparent heterogeneity in tumor microvascular perfusion (16, 32). The US contrast agent is a blood pool contrast agent that can improve the contrast between the tumor and the surrounding tissues because it cannot penetrate the intercellular space into the interstitial space (33, 34). Therefore, CEUS radiomics can better capture the vascular characteristics of different breast cancer subtypes, which is beneficial to the classification. However, the advantage of CEUS radiomics was not reflected in predicting Luminal B breast cancer and TNBC; the reason could be that Luminal B includes both HER2-positivity and HER2-negativity subtypes, which may overlap with other non-Luminal B in the characteristics of HER2 expression; hence, its CEUS performance is not easy to distinguish. TNBC mostly had a swollen appearance, and its CUS and CEUS findings revealed a clear boundary with the surrounding tissues; therefore, there may be not much auxiliary information from CEUS compared with that from CUS.

This study does have some limitations. To better evaluate the distribution of tumor vasculature, the ROI of the CEUS videos was drawn with a rectangular box, and therefore, some of the shape features of the tumor were lost. There was also some imbalanced sample size between molecular subtype categories of breast cancer, especially in the category of HER2-overexpression breast cancer and TNBC. In the research process of radiomics, the standardization of medical imaging data collection is a key problem that limits its clinical reproducibility (35). Moreover, Lee et al. found that there is no statistically significant difference in radiomics scores of the breast tumor obtained by different US machines (Philips IU22, HDI5000, and GE9), indicating that US radiomics has a general application (8). Therefore, further supporting findings from multicenter, prospective, and large-sample-size studies are needed in the future.

## Conclusions

CUS radiomics has the potential to diagnose breast cancer and predict its molecular subtype. On this basis, CEUS video can help CUS radiomics model improve its predictive performance, which provides a new idea for a non-invasive diagnosis and prediction of molecular subtype of breast cancer before surgery.

## Data availability statement

The raw data supporting the conclusions of this article will be made available by the authors, without undue reservation.

## Ethics statement

The studies involving human participants were reviewed and approved by the independent ethics committee of Cancer Hospital, Chinese Academy of Medical Sciences and Peking Union Medical College. The patients/participants provided their written informed consent to participate in this study.

## Author contributions

XG, conceptualization, methodology, formal analysis, and writing - original draft. QL, methodology, data curation, and writing - review and editing. LG, conceptualization and investigation. CC, methodology and software. XL, methodology, writing – review, and editing. XZ and DY, investigation. BW and CS, resources. LL, conceptualization, writing - review and editing, supervision. YW, conceptualization, writing - review and editing, supervision, funding acquisition. All authors contributed to the article and approved the submitted version.

## References

[1] Sung H Ferlay J Siegel RL Laversanne M Soerjomataram I Jemal A. Global cancer statistics 2020: GLOBOCAN estimates of incidence and mortality worldwide for 36 cancers in 185 countries. CA Cancer J Clin (2021) 71(3):209–49. doi: 10.3322/caac.21660 33538338

[2] Goldhirsch A Wood WC Coates AS Gelber RD Thürlimann B Senn HJ. Strategies for subtypes–dealing with the diversity of breast cancer: highlights of the st. gallen international expert consensus on the primary therapy of early breast cancer 2011. Ann Oncol (2011) 22(8):1736–47. doi: 10.1093/annonc/mdr304 PMC3144634 21709140

[3] Britt KL Cuzick J Phillips KA. Key steps for effective breast cancer prevention. Nat Rev Cancer (2020) 20(8):417–36. doi: 10.1038/s41568-020-0266-x 32528185

[4] Phung MT Tin Tin S Elwood JM. Prognostic models for breast cancer: a systematic review. BMC Cancer. (2019) 19(1):230. doi: 10.1186/s12885-019-5442-6 30871490 PMC6419427

[5] Yip SS Aerts HJ. Applications and limitations of radiomics. Phys Med Biol (2016) 61(13):R150–66. doi: 10.1088/0031-9155/61/13/R150 PMC4927328 27269645

[6] Lambin P Rios-Velazquez E Leijenaar R Carvalho S van Stiphout RG Granton P. Radiomics: extracting more information from medical images using advanced feature analysis. Eur J Cancer (2012) 48(4):441–6. doi: 10.1016/j.ejca.2011.11.036 PMC4533986 22257792

[7] Aerts HJ Velazquez ER Leijenaar RT Parmar C Grossmann P Carvalho S. Decoding tumour phenotype by noninvasive imaging using a quantitative radiomics approach. Nat Commun (2014) 5:4006. doi: 10.1038/ncomms5006 24892406 PMC4059926

[8] Lee SE Han K Kwak JY Lee E Kim EK. Radiomics of US texture features in differential diagnosis between triple-negative breast cancer and fibroadenoma. Sci Rep (2018) 8(1):13546. doi: 10.1038/s41598-018-31906-4 30202040 PMC6131410

[9] Gillies RJ Kinahan PE Hricak H. Radiomics: images are more than pictures, they are data. Radiology (2016) 278(2):563–77. doi: 10.1148/radiol.2015151169 PMC4734157 26579733

[10] Limkin EJ Sun R Dercle L Zacharaki EI Robert C Reuzé S. Promises and challenges for the implementation of computational medical imaging (radiomics) in oncology. Ann Oncol (2017) 28(6):1191–206. doi: 10.1093/annonc/mdx034 28168275

[11] Valdora F Houssami N Rossi F Calabrese M Tagliafico AS. Rapid review: radiomics and breast cancer. Breast Cancer Res Treat (2018) 169(2):217–29. doi: 10.1007/s10549-018-4675-4 29396665

[12] Wan CF Du J Fang H Li FH Zhu JS Liu Q. Enhancement patterns and parameters of breast cancers at contrast-enhanced US: correlation with prognostic factors. Radiology (2012) 262(2):450–9. doi: 10.1148/radiol.11110789 22282183

[13] Wan CF Liu XS Wang L Zhang J Lu JS Li FH. Quantitative contrast-enhanced ultrasound evaluation of pathological complete response in patients with locally advanced breast cancer receiving neoadjuvant chemotherapy. Eur J Radiol (2018) 103:118–23. doi: 10.1016/j.ejrad.2018.04.005 29803376

[14] Zhao YX Liu S Hu YB Ge YY Lv DM. Diagnostic and prognostic values of contrast-enhanced ultrasound in breast cancer: a retrospective study. Onco Targets Ther (2017) 10:1123–9. doi: 10.2147/OTT.S124134 PMC5328613 28260926

[15] Pinker K Chin J Melsaether AN Morris EA Moy L. Precision medicine and radiogenomics in breast cancer: new approaches toward diagnosis and treatment. Radiology (2018) 287(3):732–47. doi: 10.1148/radiol.2018172171 29782246

[16] Li H Zhu Y Burnside ES Huang E Drukker K Hoadley KA. Quantitative MRI radiomics in the prediction of molecular classifications of breast cancer subtypes in the TCGA/TCIA data set. NPJ Breast Cancer (2016) 2:16012. doi: 10.1038/npjbcancer.2016.12 27853751 PMC5108580

[17] Ma W Zhao Y Ji Y Guo X Jian X Liu P. Breast cancer molecular subtype prediction by mammographic radiomic features. Acad Radiol (2019) 26(2):196–201. doi: 10.1016/j.acra.2018.01.023 29526548 PMC8082943

[18] Aide N Salomon T Blanc-Fournier C Grellard JM Levy C Lasnon C. Implications of reconstruction protocol for histo-biological characterisation of breast cancers using FDG-PET radiomics. EJNMMI Res (2018) 8(1):114. doi: 10.1186/s13550-018-0466-5 30594961 PMC6311169

[19] Goldhirsch A Winer EP Coates AS Gelber RD Piccart-Gebhart M Thürlimann B. Personalizing the treatment of women with early breast cancer: highlights of the St gallen international expert consensus on the primary therapy of early breast cancer 2013. Ann Oncol (2013) 24(9):2206–23. doi: 10.1093/annonc/mdt303 PMC3755334 23917950

[20] Zhang K Zhang L Yang MH. Fast compressive tracking. IEEE Trans Pattern Anal Mach Intell (2014) 36(10):2002–15. doi: 10.1109/TPAMI.2014.2315808 26352631

[21] Peng H Long F Ding C. Feature selection based on mutual information: criteria of max-dependency, max-relevance, and min-redundancy. IEEE Trans Pattern Anal Mach Intell (2005) 27(8):1226–38. doi: 10.1109/TPAMI.2005.159 16119262

[22] Youk JH Kwak JY Lee E Son EJ Kim JA. Grayscale ultrasound radiomic features and shear-wave elastography radiomic features in benign and malignant breast masses. Ultraschall Med (2020) 41(4):390–6. doi: 10.1055/a-0917-6825 31703239

[23] Luo WQ Huang QX Huang XW Hu HT Zeng FQ Wang W. Predicting breast cancer in breast imaging reporting and data system (BI-RADS) ultrasound category 4 or 5 lesions: a nomogram combining radiomics and BI-RADS. Sci Rep (2019) 9(1):11921. doi: 10.1038/s41598-019-48488-4 31417138 PMC6695380

[24] Li Y Liu Y Zhang M Zhang G Wang Z Luo J. Radiomics with attribute bagging for breast tumor classification using multimodal ultrasound images. J Ultrasound Med (2020) 39(2):361–71. doi: 10.1002/jum.15115 31432552

[25] Wu J Li B Sun X Cao G Rubin DL Napel S. Heterogeneous enhancement patterns of tumor-adjacent parenchyma at MR imaging are associated with dysregulated signaling pathways and poor survival in breast cancer. Radiology (2017) 285(2):401–13. doi: 10.1148/radiol.2017162823 PMC5673053 28708462

[26] Kim SA Cho N Ryu EB Seo M Bae MS Chang JM. Background parenchymal signal enhancement ratio at preoperative MR imaging: association with subsequent local recurrence in patients with ductal carcinoma *in situ* after breast conservation surgery. Radiology (2014) 270(3):699–707. doi: 10.1148/radiol.13130459 24126372

[27] Guo Y Hu Y Qiao M Wang Y Yu J Li J. Radiomics analysis on ultrasound for prediction of biologic behavior in breast invasive ductal carcinoma. Clin Breast Cancer (2018) 18(3):e335–44. doi: 10.1016/j.clbc.2017.08.002 28890183

[28] Yu FH Wang JX Ye XH Deng J Hang J Yang B. Ultrasound-based radiomics nomogram: a potential biomarker to predict axillary lymph node metastasis in early-stage invasive breast cancer. Eur J Radiol (2019) 119:108658. doi: 10.1016/j.ejrad.2019.108658 31521878

[29] Marino MA Pinker K Leithner D Sung J Avendano D Morris EA. Contrast-enhanced mammography and radiomics analysis for noninvasive breast cancer characterization: initial results. Mol Imaging Biol (2020) 22(3):780–7. doi: 10.1007/s11307-019-01423-5 PMC7047570 31463822

[30] Bianchini G De Angelis C Licata L Gianni L. Treatment landscape of triple-negative breast cancer - expanded options, evolving needs. Nat Rev Clin Oncol (2022) 19(2):91–113. doi: 10.1038/s41571-021-00565-2 34754128

[31] Braicu C Raduly L Morar-Bolba G Cojocneanu R Jurj A Pop LA. Aberrant miRNAs expressed in HER-2 negative breast cancers patient. J Exp Clin Cancer Res (2018) 37(1):257. doi: 10.1186/s13046-018-0920-2 30342533 PMC6196003

[32] Zhang XY Cai SM Zhang L Zhu QL Sun Q Jiang YX. Association between vascular index measured *via* superb microvascular imaging and molecular subtype of breast cancer. Front Oncol (2022) 12:861151. doi: 10.3389/fonc.2022.861151 35387128 PMC8979674

[33] Zhao P Zhu J Wang L Li N Zhang XH Li JF. Comparative diagnostic performance of contrast-enhanced ultrasound and dynamic contrast-enhanced magnetic resonance imaging for differentiating clear cell and non-clear cell renal cell carcinoma. Eur Radiol (2023) 33(5):3766–74. doi: 10.1007/s00330-023-09391-9 36725722

[34] Guo R Lu G Qin B Fei B. Ultrasound imaging technologies for breast cancer detection and management: a review. Ultrasound Med Biol (2018) 44(1):37–70. doi: 10.1016/j.ultrasmedbio.2017.09.012 29107353 PMC6169997

[35] Lambin P Leijenaar RTH Deist TM Peerlings J de Jong EEC van Timmeren J. Radiomics: the bridge between medical imaging and personalized medicine. Nat Rev Clin Oncol (2017) 14(12):749–62. doi: 10.1038/nrclinonc.2017.141 28975929

